# Vertigo Worse from Motion

**Published:** 1896-06

**Authors:** C. M. Boger

**Affiliations:** Parkersburg, W. Va.


### VERTIGO WORSE FROM MOTION.

C. M. Boger, M. D., Parkersburg, W. Va.

In the December number of The Homœopathic Physician, at page 563, I saw a case reported by Dr. Rufus Thurston of symptoms produced by Morphine. Among these symptoms I particularly noticed the symptom " Vertigo from the least motion of the head." A case occurred in my own practice of vertigo from the least motion of the head.

Miss M., a young lady twenty-two years old, dark, spare build. Headache with sensation of being wound up tight. Sleepiness and numbness of the lower extremities. Intense vertigo on least motion of the head. ℞ Morphia-sulph.$^{cm}$, and the next day all the symptoms were gone. This prescription was made upon the statement of Dr. Thurston in the article to which I have referred.

After this successful prescription I received the March number of The Homœopathic Physician and saw in that number, at page 123, a confirmation of this same symptom by Dr. Thomas Skinner, of London, England.

